# Dr. Robert N. Blanchard

**Published:** 1918-03

**Authors:** 


**Dr. Robert N. Blanchard,** Buffalo 1888, died in Jamestown, Jan. 19, age 62.

